# White matter tracts characteristics in habitual decision-making circuit underlie ritual behaviors in anorexia nervosa

**DOI:** 10.1038/s41598-021-95300-3

**Published:** 2021-08-05

**Authors:** Reza Tadayonnejad, Fabrizio Pizzagalli, Stuart B. Murray, Wolfgang M. Pauli, Geena Conde, Ausaf A. Bari, Michael Strober, John P. O’Doherty, Jamie D. Feusner

**Affiliations:** 1 Division of Neuromodulation, Semel Institute for Neuroscience and Human Behavior, University of California at Los Angeles, Room # 57.464, 760 Westwood Plaza, Los Angeles, CA 90024 USA; 2 Division of Humanities and Social Sciences, California Institute of Technology, Pasadena, CA USA; 3 Imaging Genetics Center, Mark and Mary Stevens Neuroimaging and Informatics Institute, Keck School of Medicine of USC, Marina del Rey, CA USA; 4 Department of Neuroscience “Rita Levi Montalcini”, University of Turin, Turin, Italy; 5 Department of Psychiatry and the Behavioral Sciences, University of Southern California, Los Angeles, CA USA; 6 Artificial Intelligence Platform, Redmond, WA USA; 7 Division of Cognitive Neuroscience, Semel Institute for Neuroscience and Human Behavior, University of California at Los Angeles, Los Angeles, CA USA; 8 Department of Neursurgery, University of California at Los Angeles, Los Angeles, CA USA; 9 Division of Child and Adolescent, Semel Institute for Neuroscience and Human Behavior, University of California at Los Angeles, Los Angeles, CA USA; 10 Computation and Neural Systems Program and Division of Humanities and Social Sciences, California Institute of Technology, Pasadena, CA USA; 11 Centre for Addiction and Mental Health, Toronto, ON Canada; 12 Department of Psychiatry, Division of Neurosciences and Clinical Translation, University of Toronto, Toronto, ON Canada

**Keywords:** Diseases, Psychiatric disorders

## Abstract

Anorexia nervosa (AN) is a difficult to treat, pernicious psychiatric disorder that has been linked to decision-making abnormalities. We examined the structural characteristics of habitual and goal-directed decision-making circuits and their connecting white matter tracts in 32 AN and 43 healthy controls across two independent data sets of adults and adolescents as an explanatory sub-study. Total bilateral premotor/supplementary motor area-putamen tracts in the habit circuit had a significantly higher volume in adults with AN, relative to controls. Positive correlations were found between both the number of tracts and white matter volume (WMV) in the habit circuit, and the severity of ritualistic/compulsive behaviors in adults and adolescents with AN. Moreover, we found a significant influence of the habit circuit WMV on AN ritualistic/compulsive symptom severity, depending on the preoccupations symptom severity levels. These findings suggest that AN is associated with white matter plasticity alterations in the habit circuit. The association between characteristics of habit circuit white matter tracts and AN behavioral symptoms provides support for a circuit based neurobiological model of AN, and identifies the habit circuit as a focus for further investigation to aid in development of novel and more effective treatments based on brain-behavior relationships.

## Introduction

Anorexia nervosa (AN) is a debilitating, chronic, and potentially life-threatening psychiatric disorder, which is characterized by self-imposed starvation, physical emaciation, an intense fear of weight gain, and a profound disturbance in the way one’s shape and weight is experienced^1^. The persistence of driven weight loss behaviors in AN, despite severe emaciation, medical instability, and often numerous intensive specialized treatments, is well documented^2^. Consequently, AN is characterized among mental illnesses with the highest mortality rates^2^. One study showed that more than half of those afflicted still meet diagnostic criteria more than two decades after illness onset^3^. Despite decades of controlled treatment trials, the efficacy of existing treatments remains limited^4^ and the pathophysiology remains elusive. Critical to enhancing treatment outcomes in AN is a comprehensive understanding of its neurobiology, and it is broadly accepted that treatments for AN can ‘no longer remain brainless’^5^.

Of particular importance are the mechanisms underpinning decision-making in AN. The enigmatic persistence of habitual symptomatic behaviors such as dietary restriction, compulsive exercising, and food-related rituals, even beyond weight normalization, has raised an important dialogue around the centrality of goal-directed versus habit-based decision making in AN^6^. In the context of a dual-system of behavioral control, habit- and goal-directed systems may be conceptualized as competing systems that interact to control behavior^7^. Goal-directed behaviors are processed “reflectively” and “prospectively” by considering possible actions and their likely consequent outcomes, in contrast to habitual actions which are performed “reflexively” and “retrospectively” by relying on previous experiences of executed actions (responses) and their observed good or bad outcomes^7–9^. A theory posits that the behavioral symptoms in many with AN originally manifest as goal-directed weight loss behavior, which is operantly maintained by weight loss; over time and with significant repetition, these become habit-based, persisting irrespective of the presence of rewards, and even in the context of potentially lethal medical sequelae^10,11^ . Indeed, those with AN generally demonstrate high self-reported habit strength, relative to healthy controls^12^. Moreover, self-reported habit strength appears to be linearly related to illness chronicity and severity in those with AN and may uniquely and reliably explain variance in AN symptom severity^12,13^.

**Figure 1:**
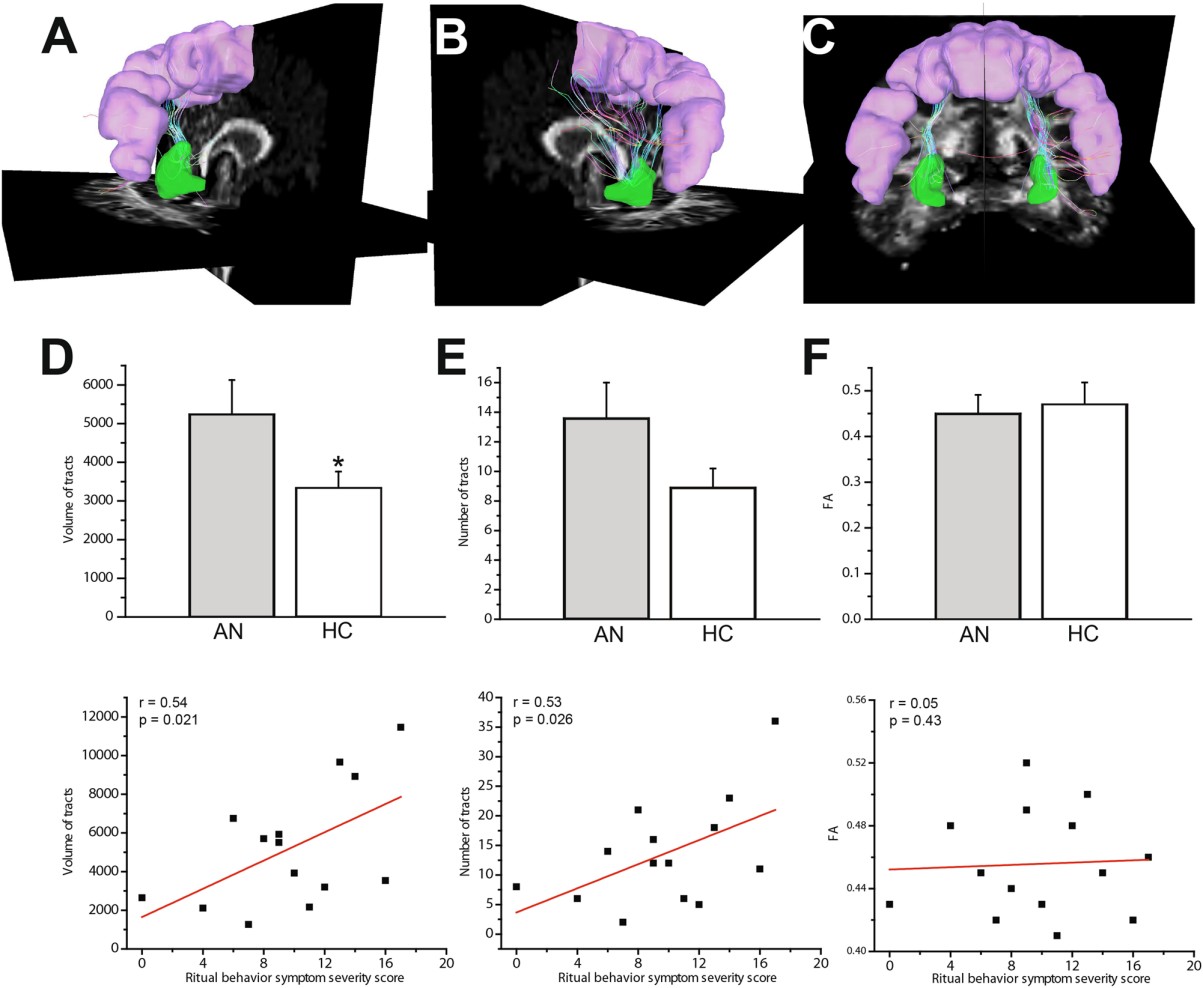
White matter tracts in the habit circuit. Views of the right (**A**), left (**B**) and bilateral (**C**) premotor/SMA (pink)-posterolateral putamen (green) tracts in an example healthy participant. (**D**) Mean volume of total bilateral premotor/SMA-putamen tracts was significantly higher in the adult AN group compared to the healthy controls (* indicates *P* < 0.05) and showed significant correlation with ritual behavior symptom severity (ritual subscale of the Yale-Brown-Cornell Eating Disorder Scale). (**E**) Compared to the healthy controls, the adult AN group shows a near significant higher bilateral tracts number. Moreover, the tracts number was found to have a significant correlation with ritual behavior symptom severity. (**F**) FA value in the bilateral premotor/SMA-putamen tracts was not found to be significantly different between AN and HC groups and did not show significant correlation with ritual behavior symptom severity.

**Figure 2:**
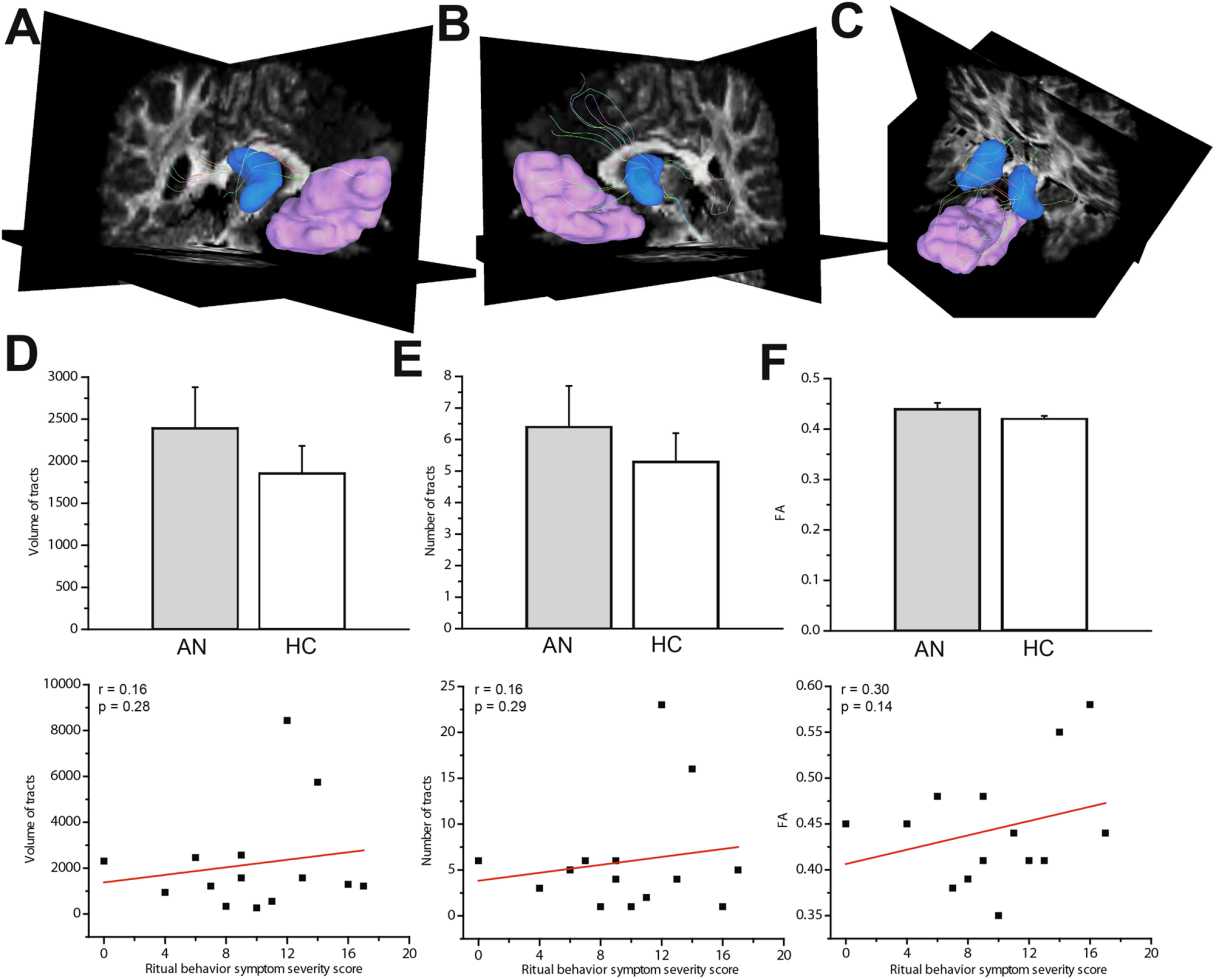
White matter tracts in the goal-directed circuit. Views of the right (**A**), left (**B**) and bilateral (**C**) anterior caudate (blue)-vmPFC (pink) tracts in an example healthy participant. Mean volume (**D**), number of tracts (**E**) and FA (**F**) in vmPFC-caudate tract streamlines did not significantly differ between adult AN and HC. There were no significant correlations between any of values and ritual behavior symptom severity.

Mechanistically, the neural substrates of goal-directed and habitual behavior control systems differ and are spatially separated. Lesion studies in rodents and functional magnetic resonance imaging (fMRI) research in humans point towards distinct underlying functional anatomy subserving goal-directed and habitual systems^14^. The medial prefrontal/medial orbitofrontal cortex (vmPFC) (prelimbic cortex in rats) and caudate (dorsomedial striatum in rats) are implicated in goal-directed actions, whereas the posterior lateral putamen (dorsolateral striatum in rats) and supplementary motor area (SMA) are modulatory in habitual actions^14–17^. Within each system, the major nodes are functionally and structurally connected, comprising distinct habit (the posterior lateral putamen-SMA, Fig. 1A–C) and goal-directed (anterior caudate-vMPFC, Fig. 2A–C) behavioral control circuits, respectively^18–22^. From the interrogation of goal-directed versus habitual decision making in a disorder non-congruent reward processing paradigm in those with AN, a bimodal distribution of habit versus goal-directed behavioral control was recently reported^6^. However, fMRI data suggest no abnormal neural activity, relative to healthy controls, underpinning either habit or goal directed behavioral circuitry^6^. In the context of disorder-congruent tasks, however, fMRI studies suggest that food choice, and the avoidance of high-fat foods in those with AN, is marked by reliably elevated dorsal striatal activity^23^, which has been interpreted to reflect elevated habit control. Moreover, in this study functional connectivity between the dorsal striatum and dorsolateral prefrontal cortex was negatively associated with actual food intake in a lab setting, suggesting that maladaptive food choices in AN may implicate circuitry underlying habitual behavior^23^. Alongside these mixed results from fMRI studies, a noteworthy absence in the current literature in AN is any examination of white matter tracts related to habitual behaviors.

In healthy individuals, the gray matter density of the putamen and the strength (number) of the white matter tracts that connect it to the premotor region are associated with the propensity for habitual behavioral control during an instrumental learning-based choice selection task^24^. Furthermore, this study found that the strength of white matter tracts connecting the caudate and vmPFC regions was associated with a tendency for goal-directed responding during the performance of the same task. VBM studies in AN have mainly reported the well-described phenomenon of *global* gray matter volume reduction in both adults^25^ and adolescents^25,26^ with AN. However, findings of *regional* gray matter volume abnormalities in AN are less consistent. A meta-analysis of 7 VBM studies (mixed adults and adolescents) reported a decrease in gray matter volume in the right caudate (although not specifically the anterior caudate involved in goal-directed decision-making) in individuals with AN compared to controls^27^. Two studies also showed a reduction in SMA gray matter volume^28,29^.

In the current study, we used diffusion tensor imaging, voxel-based morphometry, and gray matter volumes to examine, firstly, the connecting white matter tracts of habit circuits and the gray matter density and volumes of the associated regions in a group of weight-restored adults with AN compared to healthy controls of equivalent age. Secondly, as an exploratory follow-up analysis and an examination in a cohort earlier in their course of illness, we repeated the same analysis in a separately-acquired group of adolescents with AN and controls of equivalent age. Our primary a priori rationale for investigating an adolescent group alongside adult AN individuals is based on the theoretical model of AN suggesting that the symptomatic behaviors initially have a goal-directed nature, whereby weight loss is pursued as personal goals, by performing repetitive behaviors such as dieting or over-exercising^10^. Subsequently, and with excessive repetition, those assumed goal-directed behaviors are theorized to become increasingly more automatic or habitual, triggered by food and body weight/shape-related internal preoccupations or external cues^10^.

Under the aegis of the previously mentioned framework of the habit model of anorexia, we have two sets of a priori hypotheses: (a) we hypothesized that gray matter hypertrophy, gray matter higher density, and stronger structural connectivity in the habit circuit, specifically the SMA and putamen, would be evident in adults with AN with lesser or minimal changes in the adolescents with AN, (b) We also hypothesized that structural and white matter connectivity abnormalities in the habit circuit would correlate with the severity of ritualistic AN symptoms. In our first set of exploratory analyses, we indexed gray matter and white matter tract characteristics within the goal-directed circuit (ventromedial prefrontal cortex [vmPFC] and caudate) for all participants and examined their associations with AN symptoms. In our second exploratory post-hoc analysis, we performed a set of mediation/moderation analyses exploring an AN behavior- (ritual) dependent white matter “plasticity model” by modeling the influence of the characteristics of tracts in habitual decision-making circuit upon ritual severity and exploring the potential mediating or moderating role of preoccupation severity.

## Methods

### Patient recruitment

This study comprised two separate sub-studies. In the first sub-study with adult participants (Adult study), 50 individuals participated: female weight-restored AN (Adult AN; n = 20), and age-equivalent healthy controls (HC; Adult HC; n = 30; 25 females and five males). In the second sub-study, 25 female adolescent participants (Adolescent study) were recruited: adolescents with AN (Adolescent AN; n = 12) and an age-equivalent adolescent control group (Adolescent HC; n = 13). Recruitment for adult AN was undertaken via local specialized treatment centers, online and community-based advertisements, and campus flyers. For the adult sub-study, we excluded any participants if they met the following criteria: (1) pregnancy; (2) ferromagnetic parts in their bodies; (3) heavier than 280 lbs.; (4) psychiatric medications within 8 weeks before enrolling in the study or currently undergoing cognitive behavioral therapy; (5) neurological disorder or any medical condition that may affect cerebral metabolism; (6) MADRS score > 44; (7) BMI < 18.5. Our exclusion criteria for adult participants with AN included: (1) current or past comorbidity with body dysmorphic disorder; (2) any Axis I disorder other than dysthymia, major depressive disorder, panic disorder, agoraphobia, generalized anxiety disorder, or social phobia. These are highly common comorbidities in AN and it would not be a representative sample to exclude them. We excluded controls who had any Axis I diagnosis. Inclusion criteria for AN group were: (1) meet criteria for AN diagnosis; (2) be weight restored (BMI > 18.5).

Adolescents with AN and their healthy controls were part of a separate sub-study and had different inclusion/exclusion criteria. Inclusion criteria for AN were: (1) Ages 10–19; (2) Met DSM-5 criteria for Anorexia Nervosa, Restricting Type, within the previous 6 months; (3) Completed treatment as usual in an inpatient, residential, or partial hospitalization program (2–5 times/week) consisting of psychotherapy and dietary monitoring, within the previous 3 weeks; (4) Participants may be unmedicated or be taking a serotonin reuptake inhibitor medication at a stable dose for at least eight weeks at the time of enrollment. They may not be taking any other psychotropic medication, excluding a short half-life sedative/hypnotic for insomnia or a short half-life benzodiazepine as needed for anxiety but not exceeding a frequency of 3 doses in one week and not to be taken on the days of the scans. Exclusion criteria for adolescent participants with AN include (1) Lifetime Axis I bipolar disorder, lifetime psychotic disorders, lifetime ADHD, or current post-traumatic stress disorder; (2) Pathological gambling, as assessed with the South Oaks Gambling Screen; (3) Current neurological disorder; (4) Pregnancy; (5) Current major medical disorders that may affect cerebral metabolisms such as diabetes or thyroid disorders; (6) Current risk of suicide with a plan and intent; (7) A Children’s Depression Rating Scale-Revised (CDRS-R) score > 75(extremely ill) or major depressive disorder with psychotic features; (8) Ferromagnetic metal implantations or devices (electronic implants or devices, infusion pumps, aneurysm clips, metal fragments or foreign bodies, metal prostheses, joints, rods or plates); (9) Adjusted BMI ≥ 25 (overweight); (10) Visual acuity worse than 20/35 for each eye as determined by Snell enclose vision chart. Acuity may be met with corrective lenses. Inclusion criteria for control participants were: (1) Females ages 10–19; (2) score at least 0.5 standard deviations higher than population norms on the anxiety portion of the Depression Anxiety Stress Scale (DASS-21). Exclusion criteria for control participants were: (1) Any DSM-5 diagnosis (on the MINI); (2) Taking any psychiatric medication. Exclusion criteria for all participants were: (1) current substance abuse or dependence, including nicotine; (2) pathological gambling, as assessed with the South Oaks Gambling Screen; (3) current neurological disorder; (4) pregnancy; (5) current major medical disorders that may affect cerebral metabolisms such as diabetes or thyroid disorders; (6) current risk of suicide with a plan and intent; (7) a Children’s Depression Rating Scale Revised (CDRS-R) score > 75 (extremely ill) or major depressive disorder with psychotic features; (8) ferromagnetic metal implantations or devices (electronic implants or devices, infusion pumps, aneurysm clips, metal fragments or foreign bodies, metal prostheses, joints, rods or plates); (9) adjusted BMI ≥ 25 (overweight) (10) visual acuity worse than 20/35 for each eye as determined by Snellen close vision chart. Acuity may be met with corrective lenses. Adolescent participants with AN were recruited from the inpatient eating disorder unit at UCLA and from local treatment centers; they were enrolled at the end of their treatment in these settings when they met each treatment center’s individual criteria for transitioning to a lower level of care. As such, they were either partially or fully weight-restored.

The UCLA Institutional Review Board approved the study’ protocols and all methods were performed in accordance with the UCLA Institutional Review Board guidelines and regulations. All participants provided written informed consent. For adolescent study, informed consents were obtained from parents and/or legal guardians. Clinical evaluations of all participants with AN were performed by licensed psychiatrists or psychologists with clinical experience with this population. Primary or comorbid diagnoses were screened with the Mini-International Neuropsychiatric Interview (MINI v. 6.0)^30^. The Hamilton Anxiety Rating Scale (HAMA) and Montgomery–Åsberg Depression Rating Scale, widely used and well-validated clinician-rated scale for measuring anxietyz^31^ and depressionz^32^, respectively, were also administered. The severity of eating- and body/weight-related preoccupations and rituals was measured by the Yale-Brown-Cornell Eating Disorder Scale (YBC-EDS)^33^. Participant responses to items assessing preoccupations and rituals, respectively, were delineated and coded as anorexic preoccupations and ritual behavior symptom severity, respectively.

### Structural and diffusion MRI data acquisition

All brain MRI data were acquired using a Siemens Trio 3 T scanner and a 12-channel head coil. High-resolution T1-weighted images were acquired with MPRAGE (Magnetization Prepared Rapid Acquisition Gradient Echo) sequence (Slice thickness = 0.80 mm; Base resolution 320; Phase resolution 100%; Slice resolution 100%). Diffusion-weighted MRI (dMRI) data were acquired using single-shot spin-echo echo-planar imaging (EPI) (field of view = 240 mm; voxel size = 2.5 × 2.5 × 3.0 mm (1.5 × 1.5 × 1.5 mm in Adolescent study), with 0.75 mm gap; TR/TE = 7400/96 ms (3222/89 ms in Adolescent study) and flip angle 9° (89° in Adolescent study). We collected 44 (92 in Adolescent study) slices contiguous axial slices aligned to the AC-PC line along 34 gradient-sensitizing directions with b = 1000 s/mm^2^ (3000 s/mm^2^ in adolescent study) and one minimally diffusion-weighted scan.

### Region-of-interest selection

As both the premotor and SMA are functionally and structurally connected to the posterolateral putamen (Put), premotor areas of 55b, 6ma, 6d, 6mp, 6v, 6r, 6a from the HCP-MMP1 atlas^34^ were merged with “supp-motor-area” region from the AAL atlas^35^ to create a combined premotor/SMA ROI. For the vmPFC ROI, 10r, 10v, OFC regions from the HCP-MMP1 atlas were merged with “Frontal_Med_orb” region from the AAL atlas. Posterolateral putamen (Put) and anterior caudate (Cau) ROIs were selected from Pauli et al., striatum atlas^36^.

### Voxel-based morphometry analysis (VBM) for density and volumetric analysis

Gray matter density maps were obtained using a VBM framework. Briefly, T1-weighted volumes were classified into gray matter (GM), white matter (WM), cerebrospinal fluid (CSF) and non-brain tissue using the ‘Unified Segmentation’ framework^37^ implemented in SPM12, running under Matlab 9.0 (Mathworks, Sherborn, MA, USA). For optimal tissue classification of subcortical regions, we adopted new tissue probability maps (TPMs) described in Lorio et al.^38^. The resulting tissue probability maps were transformed non-linearly to standard MNI space using the diffeomorphic inter-subject registration algorithm (DARTEL)^39,40^. For statistical analysis of GM density, an isotropic Gaussian smoothing kernel of 6 mm full-width-at-half-maximum (FWHM) was applied on the resulting GM maps. For volumetric analysis, a "modulation" step implemented in SPM has been applied to the GM maps before smoothing.

### Diffusion-weighted tractography

We used DSI studio (http://dsi-studio.labsolver.org) for dMRI data preprocessing, quality inspection, reconstruction, and tractography, all performed in individual native spaces. NIFTI formatted dMRI data was first converted into SRC format that includes information about diffusion-weighted volumes, image dimensions, voxel size, and the b-table. SRC files were used for quality inspection based on the consistency of image dimensions, resolution, DWI count, and neighboring DWI correlation. A DTI diffusion method was used for reconstruction that includes thresholding, smoothing, and defragmentation for removing the background noise, increasing the reconstruction efficacy, and facilitating further visualization. The in-plane resolution was 1.98 mm. The slice thickness was 2 mm. The diffusion tensor was calculated. A deterministic fiber tracking algorithm^41^ was utilized. DSI studio initially brings three atlases of HCP MMP1.0, AAL and Pauli et al. striatum to the subject native space by applying a nonlinear registration procedure. Seeds from those atlases were selected and merged (in the cases of premotor/SMA and vmPFC, please see above) to define ROIs for subsequent ROI-to-ROI fiber tacking and isolation of habit and goal-directed decision-making circuit tracts. The habit decision making circuit was defined by streamlines that connect the premotor/SMA ROI to the posterolateral putamen ROI (premotor/SMA-putamen tracts) compared to the goal-directed decision-making circuit which was defined by streamlines connecting the vmPFC ROI to the anterior caudate ROI (vmPFC-caudate tracts). The angular threshold was 60 degrees. The step size was 0.99 mm. The anisotropy threshold was determined automatically by DSI Studio. Tracks with length less than 30 mm were discarded. A total of 5000 seeds were placed. After fiber tracking, three quantitative metrics were extracted for each of habit and goal-directed decision-making circuit’ tracts: mean fractional anisotropy (FA), number of tracts and tracts volume.

### Statistical analyses

All statistical analyses were performed with SPSS (IBM SPSS Statistics for Windows, Version 24.0. Armonk, NY). Goal-directed and habitual ROIs’ volumes and densities were analyzed for between-group differences using an independent sample t-test in the adult dataset. Data were analyzed separately for the adults and adolescents as they were obtained from different sub-studies with different inclusion/exclusion criteria. For the adolescent dataset, ANCOVA was applied for comparing those values between AN adolescent subjects and control group while controlling for age and Pubertal Development Scale score. We did not control for ROIs gray matter volumes when comparing white matter tracts’ metrics for adult group for two reasons. First, the values of ROI gray matter volumes were not significantly different between AN and control groups in the adult study. Second, it is unlikely that the *volume* of two ROIs connected by direct white matter tracts can have a notable impact on the biophysical characteristics of the connecting tracts. For the adolescent study, ANCOVA was used for comparing white matter tracts’ metrics between AN adolescent subjects and control group while controlling for age, Pubertal Development Scale score and volumes of premotor/SMA and vmPFC regions. Pearson correlation was used to analyze the relationship between values of goal-directed and habitual ROIs’ volumes or tracts’ metrics and clinical values of preoccupations and ritual behavior symptom severity.

We performed normality tests for values of habit and goal-directed circuits’ white matter tract variables using the Kolmogorov–Smirnov test. In the adult sub-study, values of volume and number of tracts in habit circuit were found to not be normally distributed (positively skewed). In the adolescent sub-study, values of habit circuit number of tracts were detected to not be normally distributed (positively skewed). Thus, we applied log transformation of the skewed data. Log-transformed values were used to perform t-test, Pearson correlation, and ANOVA. We reported the original (non-transformed) values for means ± STD in the results section and figures for more straightforward interpretation, but the P, F, t, and r values reported in the results section are all from the stats that we reran with the log-transformed data.

We did not correct for multiple comparisons based on the rationale that the nature of our three main measures of structural connectivity in this study is ontologically distinct and different. Specifically, we were working with the following three distinct mechanistic models: the number of tracts is a neurodevelopmentally-related variable; the volume of tracts is indicative of the plasticity-based thickness of the myelin; and FA reflects the integrity of white matter tracts based on the diffusion of water molecules within axons paths. Therefore, because these are separate neurobiological measures, we did not correct across the three. For the gray matter measures, likewise, density and volumes are different neurobiological measures, so we did not correct across the two. The exploratory analyses were not corrected, due to their exploratory nature.

We only applied mediation/moderation analysis with the adult group. Owing to our a priori hypothesis (see introduction) that the nature of decision-making abnormalities in adolescent and adult subjects might be different, we did not combine the adult and adolescent data in these analyses. Mediation and moderation analyses were performed by using Hayes’s method of ordinary least squares regression-based path analysis, implemented in PROCESS macro (run in SPSS), which also includes bootstrap-based confidence interval calculations^42^. Regarding our mediation/moderation analyses (“plasticity model”) we should clarify that in any behavior-dependent plasticity phenomena, the causal dynamics between behavior and neurobiological plastic changes could be *bidirectional* and *circular*. In other words, the repetition of behavior could lead to neurobiological plastics changes in the underlying neural structures, but those very changes subsequently can cause facilitation and pronouncement of the resulting action. Because of this bidirectionality/circularity, in this plasticity model, one might test any of these biological variables (white matter tracts volume), or ritual severity, as the dependent variable. We chose ritual symptom severity as the dependent variable in the second moderation analysis because ritual behavior symptom severity in AN is the study's main focus.

## Results

### Demographics and clinical characteristics

Demographic and clinical information for adult and adolescent groups are summarized in Table 1. All participants with AN in both the adult and adolescent sub-studies were female. There are 5 male control participants in the adult sub-study. There was no significant age difference between participants with AN and their controls in either the adult or adolescent studies. As expected, participants with AN had significantly lower BMI compared to controls in both studies. Comorbidity profiles of AN adult and adolescent sub-studies are reported in Table 2. AN individuals scored higher than healthy participants on the anxiety symptom severity scale measured by the Hamilton Anxiety Scale (HAMA) in both adult and adolescent studies. In the adolescent study, where Yale-Brown-Cornell Eating Disorder Scale (YBC-EDS) was used to quantify preoccupations and rituals severity for both AN and healthy groups, the AN group showed significantly higher YBC-EDS scores compared with controls.

**Table 1:** Demographic and clinical characteristics of patients and control subjects.

	Adult AN	Adult HC	P	Adolescent AN	Adolescent HC	*P*
AGE	21 ± 4.6	22 ± 4.7	0.84	15 ± 1.8	15 ± 1.6	0.2
Gender	20 F	25 F/5 M	0.054	12 F	13 F	NaN
Pubertal Development Score	–	–	–	14.9 ± 4.3	17.7 ± 1.7	0.06
Year of Education	13.4 ± 3.33	14.0 ± 2.57	0.44	8.8 ± 1.85	10.0 ± 1.6	0.08
AN Type	16Res/4Bing	–	–	12 Res	–	–
Proportion on Medication	None	–	–	7 out of 12	–	–
BMI	19.9 ± 2.4	22 ± 3.4	0.002	23.5 ± 11.67	66.5 ± 17.99	< 0.0001
Lowest BMI	15.36 ± 1.97			13.6 ± 0.57		
Duration of illness (months)	70.0 ± 55.7			26.3 ± 24.02		
YBC-EDS	19 ± 6.5	–	–	19 ± 9.1	1.7 ± 3.3	< 0.0001
EDE Score	3.0 ± 1.36	–	–	3.1 ± 1.68	0.6 ± 0.72	< 0.0001
HAMA	6.9 ± 6.4	2 ± 1.6	0.0001	12 ± 6.9	6 ± 4.1	0.01
MADRS	11.4 ± 9.62	1.1 ± 1.6	0.0001	–	–	–
CDRS™-R	–	–	–	39.6 ± 17.20	23.3 ± 50.1	0.0001

*Abbreviations* AN: Anorexia, HC: healthy control, BMI: Body mass index (Percentile BMI values were used for Adolescent sub-study), YBC-EDS: Yale-Brown-Cornell Eating Disorder Scale, EDE: Eating Disorder Examination, HAMA, Hamilton Anxiety Scale, MADRS: Montgomery–Åsberg Depression Rating Scale, CDRS™-R: Children's Depression Rating Scale™, Revised.

**Table 2:** Comorbidities profiles of AN adult and adolescent sub-studies.

	Adolescent sub-study	Adult sub-study
Dysthymia	2	2
MDD	2	2
GAD	5	5
OCD	1	–
PTSD	1	–
PD	2	–

*Abbreviations* MDD: Major Depressive Disorder; GAD: Generalized Anxiety Disorder; OCD: Obsessive-Compulsive Disorder; PTSD: Post-Traumatic Stress Disorder; PD: Panic Disorder.

### Gray matter results

GM volumes in the premotor/SMA, putamen, vmPFC, and caudate ROIs did not significantly differ between adults with AN and their controls (Table 3). In adolescents, GM volume in premotor/SMA and vmPFC cortical areas was found to be significantly smaller in AN compared to controls. However, putamen and caudate gray matter volume did not differ between adolescent with AN and controls. GM density in the putamen and caudate ROIs did not significantly differ between AN and control groups, in both the adult or adolescent cohorts.

**Table 3:** Measures of gray matter volume and density in habit or goal-directed circuits’ regions.

	Adult AN	Adult HC	*P*	Adolescent AN	Adolescent HC	*P*
Put (GMv)	995 ± 77.2	1016 ± 120.2	0.57	1134 ± 49.0	1162 ± 63.0	0.33
Put (GMd)	0.59 ± 0.044	0.58 ± 0.046	0.85	0.57 ± 0.172	0.58 ± 0.116	0.90
premotor/SMA (MGv)	8039 ± 695.1	8305 ± 1106.6	0.43	7220 ± 463.7	8630 ± 516.5	0.001(*t* = 3.85)
Cau (GMv)	1676 ± 112.3	1690 ± 225.1	0.85	1557 ± 111.5	1694 ± 162.2	0.07
Cau (GMd)	0.62 ± 0.025	0.61 ± 0.029	0.1	0.6 ± 0.161	0.62 ± 0.123	0.60
vmPFC (MGv)	2,13,599 ± 16028.3	2,17,222 ± 30353.5	0.69	2,04,172 ± 68.0	2,19,973 ± 15824	0.02(*t* = 2.50)

*Abbreviations* AN: Anorexia, HC: healthy control, GMv: gray matter volume, GMd: gray matter density, Habit circuit regions including Put: putamen. Cau, caudate, premotor/SMA: premotor/supplementary motor area, goal-directed circuit regions including vmPFC: ventromedial prefrontal cortex.

### Tractography results

In adults with AN, total volume of bilateral tracts in the habit circuit was significantly greater in the AN group compared to the healthy controls (AN = 5241.25 ± 886.7mm^3^; HC: 3331.83 ± 425.4 mm^3^; *P* = 0.046; t = 2.05; effect size d = 0.83, Not Corrected; Fig. 1D). The total bilateral number of tract streamlines was higher in the adult AN group compared to healthy controls, at trend level (AN = 13.6 ± 2.4; HC = 8.9 ± 1.3; *P* = 0.061; t = 1.92; effect size d = 1.12, Not Corrected; Fig. 1E). In another analysis, we found that the average fractional anisotropy (FA) value in the bilateral tracts was not significantly different between adult AN and HC groups (AN = 0.45 ± 0.041; HC = 0.47 ± 0.048; F_39.900_ ; *P* = 0.20; Fig. 1F). The characteristics of the goal-directed tracts (vmPFC-caudate) did not significantly differ between adult AN and HC groups (Fig. 2D–F). In adolescents with AN, there were no significant differences in the volume (AN = 3268 ± 1628.8mm^3^; HC: 3618 ± 2367.3 mm^3^; *P* = 0.93; F = 0.006), number (AN = 13.7 ± 7.8; HC = 17.8 ± 13.12; *P* = 0.62 ; F = 0.24) , or FA (AN = 0.41 ± 0.04; HC = 0.41 ± 0.02 ; *P* = 0.85 ; F = 0.52 ; effect size d = 0.01) of premotor/SMA-putamen or vmPFC-caudate tracts, relative to controls.

### Associations with clinical variables

To examine associations with clinical variables, we performed correlation analyses, focusing on AN ritual behavior symptom severity subscale scores from the Yale-Brown-Cornell Eating Disorder Scale (YBC-EDS). These were measured in a subset of adult AN (n = 14) (the scale was introduced part-way through data collection) and adolescent AN (n = 11) subjects. The severity of ritual behavior symptoms was significantly correlated with the total bilateral volume of tracts in both the adult and adolescent AN groups (adult AN: r = 0.50, *P* = 0.033, Not Corrected**, **Fig. 1D; adolescent AN: r = 0.55, *P* = 0.037, Not Corrected). Further, the total bilateral number of tracts showed near-significant correlation with ritual behavior symptom severity scores in the adult AN group (r = 0.43; *P* = 0.058; Not Corrected; Fig. 1E) and in the adolescent AN group (r = 0.46; *P* = 0.074).

When combining the adult and adolescent AN groups (n = 25), there was also a significant association between the total bilateral tracts volume (r = 0.50; *P* = 0.005, Not Corrected) and total bilateral tracts number (r = 0.37; *P* = 0.034, Not Corrected) with ritual behavior symptom severity. Despite a strong significant correlation between preoccupations and ritual behavior symptom severity scores across participants (r = 0.59; *p* = 0.001), no significant correlation was found between preoccupation severity scores and volume (r = 0.25; *p* = 0.27) or number of the tracts (r = 0.13; 0.53). No significant correlation was found between BMI and tracts volume in adult AN group (r = − 0.25; *p* = 0.15) or adolescent group r = − 0.32; *p* = 0.16; using percentile BMI).

No significant correlation was found between the volume or number of vmPFC-caudate tracts and preoccupations or ritual behavior symptom severity scores. For the gray matter metrics, there were no significant correlations between any ROIs’ volume or gray matter density and preoccupations or ritual behavior symptom severity scores in adult or adolescent participants with AN.

We also examined the correlation between HAMA and MADRS scores and the habit circuit white matter tracts’ characteristics (post hoc analysis). We only found a significant positive correlation between habit circuit white matter tracts’ volume and HAMA score in the adult sub-study (r = 0.52, *P* = 0.02).

### Mediation and moderation results

**Figure 3:**
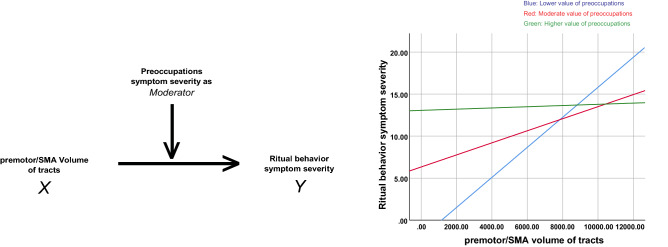
Schematic demonstration of the moderation analysis with the total premotor/SMA volume of tracts and ritual behavior symptom severity in the AN adult group. Left: ritual behavior symptom severity is the dependent variable (Y) with the total premotor/SMA volume of tracts as the independent variable (X), and the preoccupations symptom severity as the moderator. Right: graph that shows the association between X and Y among adult anorexia participants with relatively lower (blue, significant), moderate (red, significant) and higher (green, none-significant) values of anorexia nervosa-related preoccupation symptom severity.

In our post-hoc mediation/moderation analyses, we tested an AN behavior- (ritual) dependent white matter “plasticity model”, in which the volume of the tract within the habit circuit may exert influence over ritual symptom severity while examining the potential mediation or moderation role of preoccupation severity. This analysis revealed that the influence of total tracts volume of tracts (X) over AN ritual severity (Y) does indeed differ across levels of preoccupation severity (lower preoccupation severity: X effect on Y = 0.0017, *P* = 0.004, LLCI = 0.0007, ULCI = 0.0028; moderate preoccupation severity: X effect on Y = 0.0008, *P* = 0.01, LLCI = 0.0002, ULCI = 0.0013; higher preoccupation severity: X effect on Y = 0.0001, *P* = 0.86, LLCI = -0.008, ULCI = 0.009, Fig. 3). In other words, a significant habit tracts’ volume-ritual symptom severity relationship exists at the lower and moderate preoccupation symptom severity levels, but not at the higher levels.

## Discussion

Here, we assessed the biophysical characteristics of gray matter regions and connecting white matter tracts within habit and goal-directed decision-making neurocircuitries in two groups of adults and adolescents with AN. There are emerging reports of abnormal habit learning in AN^10,12^, yet no neuroimaging studies of AN to date have assessed the white matter tracts in the undergirding neural systems. The current findings in white matter tracts within the habit circuit further supports the notion of perturbations in habit-based decision-making in AN. Specifically, we found that adult AN is associated with a higher volume of white matter tracts in the habit decision-making circuit. Moreover, the volume of white matter tracts in the habit circuit was correlated with the severity of ritual behaviors in two separate anorexic adult and adolescent groups. This suggests that a circuit-based neurobiological abnormality in the habit learning system may underlie ritualistic/compulsive behaviors in this challenging to treat and potentially deadly psychiatric condition.

Our findings are in alignment with self-report data, noting (1) greater habit strength in those with AN, relative to controls, and (2) a direct correlation between habit strength and illness chronicity and severity^12^. Importantly, recent evidence suggests that therapeutic attempts to reduce habit strength in those with AN is accompanied with meaningful symptom reduction and positive increments in laboratory meals^43^, underscoring the mechanistic salience of habit-decision making in the psychopathology of AN. As of yet there has not been extensive human neurobehavioral evidence supporting this model, however, mainly due to the difficulty of experimentally manipulating AN behavior (dieting or over-exercising).

In a previous fMRI study assessing monetary reward processing in AN, behavioral response data revealed two subgroups, a “goal-directed” decision making group, and a “habit-driven” decision making group^6^. In this study, fMRI data showed that activity of the medial orbitofrontal cortex during the “reward anticipation period” was higher in habit-driven compared to the goal-directed subgroup. In a preliminary study by Godier et al., fruit and cartoon animal pictures, and noise stimuli, were used, respectively, in the slips-of-action and an avoidance paradigm to study goal-directed and habit balance in AN^44^. The study did not find any behavioral evidence of over-reliance on habit in the AN group. However, as mentioned by the authors, the study was underpowered, and it is not clear how the negative findings can be generalized to “disorder-specific” habits. Another approach to investigate habit is to utilize self-report questionnaires such as Self-Report Habit Index (SRHI). Coniglio and colleagues reported that the variability in the severity of food restriction behaviors in a group of 78 patients with anorexia could be explained meaningfully by “habit strength” (measured by SRHI), but not by cognitive restraint measures pointing to the involvement of a habit rather than cognitive control mechanism in mediating anorexic behavior^13^. Steinglass and colleagues compared habit strength in 20 individuals with AN and a healthy control group and found significantly higher habit strength values in the A group^45^. In another study, the same group successfully applied the Regulating Emotions and Changing Habits (REaCH) behavioral therapy method to reduced habit strength in hospitalized AN patients^43^. Interestingly, a reduction in habit strength was found to be accompanied by meaningful clinical eating disorder symptom improvements on the Eating Disorder Examination Questionnaire and a trend-level increase in caloric intake during the end-of-treatment laboratory meal.

To our knowledge, the present findings represent the first examination of the white matter tracts implicated in habit circuits in those with AN, and provide new insights related to abnormal habit learning and decision making in AN. Specifically, our findings suggest a stronger structural connectivity of nodes within the habit decision-making circuit in AN, as evidenced by significantly larger volume of tracts (and a near significant higher number of tracts), and in concert, significant associations between these structural connectivity metrics and AN behavior symptom severity in adults and in a separate sample of adolescents. These findings, therefore, provide novel and interesting neurobiological-based support for a habit model of AN. Interestingly, the absence of abnormalities in the gray matter and structural connectivity of the goal-directed decision making circuit in adults or adolescents with AN, and the absence of significant correlations between those metrics and habit behavior symptoms severity, suggest that decision-making perturbations in those with AN may be largely habit-based, rather than goal-directed.

Accumulating evidence from rodent and human neuroimaging studies has shown dynamic activity- and experience-dependent changes in the characteristics of white matter such as new myelin formation or change in myelin thickness, a phenomena called “white matter plasticity” that, alongside more known synaptic-based neuroplasticity mechanisms, shapes brain connections and their functional dynamics^46^. Measures of myelin thickness at the cumulative level can be roughly captured as the volume of the white matter tracts by using human neuroimaging methods. Rodents studies have shown that social isolation or neuronal stimulation can induce alteration in myelin thickness^47,48^, pointing to environmental and neural activity-dependent modulation of myelin structural features. Activity-dependent changes in volume of white matter are also reported in humans. In a longitudinal study, Colcomble et al. (2006) showed 6 months of exercise in elderly adults can cause an increase in the volume of the anterior corpus callosum^49^. In another study, the volume of tracts in the trunk and hand descending motor pathways were shown to be larger in dancers compared to a healthy control group^50^. Higher volumes of white matter have been demonstrated in other psychiatric conditions. For example, Knöchel et al., reports a significant higher volume of tracts in the fornix in schizophrenia subjects compared to healthy controls^51^. Luo et al. reported that within a group of individuals with attention-deficit/hyperactivity disorder, remitters had a significantly greater volume of right hippocampo-frontal and right parieto-insular white matter fiber tracts compared to the subgroup with no symptom remission^52^. The current finding of a larger volume of connecting white matter tracts within the habit circuit and its positive association with habit symptom severity raises the possibility that AN is associated with white matter plasticity change in the habit circuit. Based on the absence of this abnormality in the adolescent group, we can speculate that, in line with habit model of AN, white matter plasticity changes may occur in the later stage of the disorder when the anorexic behaviors become more compulsive and habitual.

Nonetheless, within the adolescent anorexic group (similar to the adult group), a significant positive correlation was found between volume of connecting fiber tracts in habit circuit and AN behavioral symptom severity. That finding suggests that even without white matter volume (plasticity) abnormalities in the habit circuit in the early stage of anorexia development, the characteristics of the connecting white matter tracts in habit circuit may have a significant role in driving AN behaviors. Although we acquired the adolescent and adult data cross-sectionally, the observation that adults but not adolescents show a larger volume of tracts in the habit circuit sets up a possible hypothesis, to be tested in a longitudinal study, that white matter volume may increase over time with the course of illness. If so, this would provide a neurobiological basis for clinical observations noting that early intervention, when habits are less well-entrenched, yields favorable outcomes^53^.

Additionally, while it has long been contested that interruption of the behavioral symptoms of AN ought to take temporal precedence to cognitive symptoms in AN, our moderation analysis findings offer some important insights on this clinical assertion. Specifically, our results suggest that the volume of the white matter tracts in the habit circuit is a significant predictor of ritual behavior severity (higher volume predicts more severe rituals). Further, while the severity of AN preoccupations plays a moderation role in that bio-behavioral (white matter volume X ritual behaviors) association, the strength of this moderation effect was weak. Thus, the modulation by AN cognitive symptoms may not have a significant impact on how the volume of white matter tracts in the habit circuit influences the severity of ritual behaviors. If so, this might underlie why there is generally more favorable symptom remission as a result of behavioral treatment approaches compared with purely cognitive treatment approaches^2^.

Regarding the significant correlation between anxiety score symptom severity and the volume of tracts in the habit circuit in adult sub-study, our interpretation is that this finding is not likely the results of *direct* interaction between anxiety and premotor/SMA-Putamen tracts, due to what is known anatomically/functionally about these tracts. Converging evidence from rodent, primate and human (lesion and neuroimaging) studies has shown that from a functional anatomical perspective, the corticostriatal premotor/SMA-Putamen connection is mainly involved in habitual behaviors and motor action^14^ and does not overlap with limbic and frontolimbic neurocircuitries that subserve anxiety and other negative emotions. Nevertheless, anxiety might have an indirect impact: the higher the level of anxiety, the higher the level of preoccupation symptom severity, causing higher ritual symptom severity, which could subsequently impact the white matter tracts’ volume over time (“plasticity model”). Alternatively, higher preoccupation symptom severity could result in higher anxiety and, in parallel, increase white matter tract volume over time. This could result in an apparent association between anxiety and white matter tract volume, although again, it would be an indirect relationship.

These findings, if replicated, may have clinical translational implications. The impact of anorexic preoccupations symptoms in facilitating the influence of the habit circuit tracts volume on ritualistic behaviors, and/or the influence of habit circuit overactivity (manifested structurally by higher volume of fiber tracts) in driving anorexic behavior, might be therapeutically reduced, e.g. with down-modulation of habit circuit functional/structural premotor/SMA connectivity. In keeping with this, one possible approach, which has been demonstrated to be efficacious in OCD treatment^54,55^, involves using inhibitory low frequency repetitive transcranial magnetic stimulation (rTMS) over premotor or SMA areas that show functional/structural connectivity with the putamen, using neuronavigation, to downregulate the hyperconnected (hyperactive) habit circuit.

This work has strengths as well as several limitations. First, the replication of findings of the significant correlation between the volume of habit circuit white matter tracts and severity of AN behavior in two separate samples supports the strength of our results. However, the adolescent sample size was limited; these findings, therefore, need to be replicated and in larger sample size studies. Second, our finding regarding evidence of the increased volume of white matter tracts in the adult but not adolescent group tested in our exploratory follow-up analysis suggests possible neurobiological sequelae of AN, which over time becomes more and more resistant to conventional treatments. However, our study was cross-sectional and adult and adolescent groups differed on other important factors besides mean age, particularly regarding weight restoration status and comorbidity profile. Also, eight out of 50 subjects in the adult sample had an age below 18. Therefore, longitudinal studies with a higher sample size are needed to temporally assess the trajectory of white matter plasticity alterations as the illness advances. Third, we performed our mediation/moderation analyses on relatively small sample size, and therefore the findings should be interpreted with caution. Nevertheless, we believe that our moderation analysis results provide valuable preliminary insights that can be utilized for testing and generation of hypotheses in future studies with larger sample sizes. Longitudinal studies are also required to make definitive causal inferences from mediation/moderation modeling. Fourth, we used a psychometrically sound and clinically widely-used measure, the YBC-EDS questionnaire administrated by clinician, which delineates various dimensions of real-life disease-related behavioral symptoms. This measure in future studies should be complemented by cognitive neuroscience-based scales of habituality measured by instrumental decision-making tasks to provide comprehensive supports of habit base model of anorexic ritual behavior. Fifth, we assessed three metrics of white matter tracts (FA, number of tracts, and tracts’ volume). Other white matter approaches such as those facilitated by diffusion spectrum imaging measures or methods such as mean diffusivity (MD), radial diffusivity (RD), or quantitative magnetization transfer (qMT) data acquisition could be used to explore other aspects of white matter pathology in habit and goal-directed circuits and their associations with clinical measures.

In sum, these findings offer important and novel insights into a circuit-based neurobiological mechanism for abnormal habit-based decision-making in AN. Our results provide evidence of white matter abnormalities in the habitual decision-making circuits in adults with AN, as well as significant associations between the characteristics of structural connectivity of the habit circuit with the severity of anorexic rituals in two independent adult and adolescent groups. Beyond the association with anorexic behaviors, subjective anorexic obsessive preoccupation was also shown to moderate the relationship between habit circuit white matter volume and compulsive rituals. The habit circuit may be a potential novel network-based neurobiological target for the treatment of maladaptive anorexic behaviors.
